# Supplementary material for: Effects of Different-Sized Cages on the Production Performance, Serum Parameters, and Caecal Microbiota Composition of Laying Hens
Source: Animals (Basel). 2023 Jan 12;13(2):266. doi: 10.3390/ani13020266 (PMC9854594; doi:10.3390/ani13020266)
Supplement: Supplementary file 1 [file animals-13-00266-s001.zip › animals-2015913-supplementary.pdf]

animals MDPI

Supplementary Materials

Effects of Different-Sized Cages on the Production Performance, Serum Parameters, and Caecal Microbiota Composition of Laying Hens

Supplementary Table S1. The relative abundance (% reads) of the most dominant phyla in the cecal microbiome of layers reared in the HC, LC, MC and SC.

Species	HC	LC	MC	SC
Bacteroidetes	44.25 ± 6.76[b]	45.99 ± 5.84[b]	34.54 ± 3.97[a]	49.84 ± 4.83[b]
Firmicutes	42.27 ± 6.25[b]	39.97 ±4.79[ab]	52.35 ± 5.62[c]	34.49 ± 5.88[a]
Actinobacteria	4.27 ± 0.53	4.94 ± 1.11	3.89 ± 0.72	3.77 ± 1.02
Proteobacteria	3.17 ± 1.1	3.50 ± 0.52	3.10 ±0 .87	3.15 ± 0.76
Euryarchaeota	1.45 ± 0.45[b]	1.57 ± 0.38[b]	0.92 ± 0.26[a]	3.01 ± 0.54[c]
Patescibacteria	1.06 ± 0.29	0.95 ± 0.21	1.93 ± 0.39	1.04 ± 0.16
Spirochaetes	1.07 ± 0.25	0.98 ± 0.14	0.84 ± 0.09	1.91 ± 0.06
Verrucomicrobia	0.23 ± 0.03	0.21 ± 0.01	0.37 ± 0.27	0.33 ± 0.02
WPS-2	0.61 ± 0.76	0.16 ± 0.08	0.41 ± 0.05	0.26 ± 0.03
Tenericutes	0.35 ± 0.29	0.38 ± 0.17	0.24 ± 0.07	0.27 ± 0.08

HC, Huge cage, LC, Lagre cage, MC, medium cage, SC, small cage. [a–c]Means with different superscripts in the same row are significantly different ($p < 0.05$).

Supplementary Table S2. The relative abundance (% reads) of the most dominant genera in the cecal microbiome of layers reared in the HC, LC, MC and SC.

Species	HC	LC	MC	SC
Bacteroides	16.45 ± 3.14[b]	16.40 ± 3.76[b]	12.04 ± 2.17[a]	18.23 ± 3.84[b]
Lactobacillus	8.91 ± 1.10[b]	6.01 ± 1.11[a]	5.91 ± 0.89[a]	8.64 ± 1.65[b]
Faecalibacterium	3.96 ± 0.75[b]	4.78 ± 0.92[b]	15.99 ± 2.14[c]	2.26 ± 0.51[a]
Prevotellaceae_UCG-001	5.14 ± 0.61	7.01 ± 1.72	5.06 ± 0.82	6.45 ± 1.55
Rikenellaceae_RC9_gut_group	6.52 ± 1.08	5.22 ± 1.42	5.55 ± 1.27	6.23 ± 1.82
Ruminococcus_torques_group	3.71 ± 0.96	3.46 ± 1.04	2.81 ± 1.62	2.80 ± 1.70
Desulfovibrio	2.58 ± 0.88	3.01 ± 0.45	2.69 ± 0.82	2.46 ± 0.68
Olsenella	1.39 ± 1.15	2.43 ±1.24	2.18 ± 0.96	1.57 ± 0.69
Methanobrevibacter	1.45 ± 0.55[a]	1.57 ± 0.58[a]	0.92 ± 0.36[a]	3.01 ± 0.94[b]
Alloprevotella	1.58 ± 0.33	1.56 ± 0.39	1.52 ± 0.58	2.14 ± 0.66

HC, Huge cage, LC, Lagre cage, MC, medium cage, SC, small cage. [a–c]Means with different superscripts in the same row are significantly different ($p < 0.05$).
